# Bidimensional Engineered Amorphous *a*-SnO_2_ Interfaces: Synthesis and Gas Sensing Response to H_2_S and Humidity

**DOI:** 10.1021/acssensors.2c00887

**Published:** 2022-06-25

**Authors:** Valentina Paolucci, Jessica De Santis, Vittorio Ricci, Luca Lozzi, Giacomo Giorgi, Carlo Cantalini

**Affiliations:** † Department of Industrial and Information Engineering and Economics, University of L’Aquila and UdR INSTM of L’Aquila, Via G. Gronchi 18, I-67100 L’Aquila, Italy; ‡ Department of Physical and Chemical Sciences, University of L’Aquila, via Vetoio, 67100 L’Aquila (AQ), Italy; § Department of Civil & Environmental Engineering (DICA), Università degli Studi di Perugia, Via G. Duranti 93, 06125 Perugia, Italy; ∥ CNR-SCITEC, 06123 Perugia, Italy

**Keywords:** SnSe_2_, thermal oxidation, amorphous SnO_2_, H_2_S, water vapor, cross-influence, DFT, mechanism

## Abstract

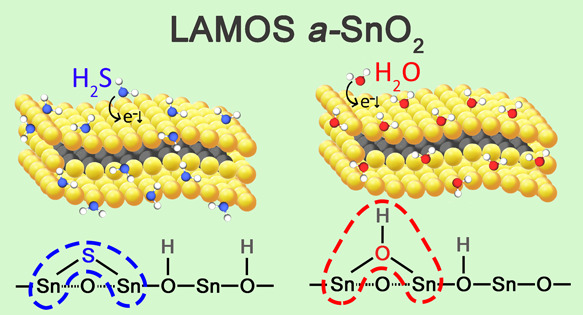

Two-dimensional (2D) transition metal dichalcogenides (TMDs) and metal chalcogenides (MCs), despite their excellent gas sensing properties, are subjected to spontaneous oxidation in ambient air, negatively affecting the sensor’s signal reproducibility in the long run. Taking advantage of spontaneous oxidation, we synthesized fully amorphous *a*-SnO_2_ 2D flakes (≈30 nm thick) by annealing in air 2D SnSe_2_ for two weeks at temperatures below the crystallization temperature of SnO_2_ (*T* < 280 °C). These engineered *a*-SnO_2_ interfaces, preserving all the precursor’s 2D surface-to-volume features, are stable in dry/wet air up to 250 °C, with excellent baseline and sensor’s signal reproducibility to H_2_S (400 ppb to 1.5 ppm) and humidity (10–80% relative humidity (RH)) at 100 °C for one year. Specifically, by combined density functional theory and ab initio molecular dynamics, we demonstrated that H_2_S and H_2_O compete by dissociative chemisorption over the same *a*-SnO_2_ adsorption sites, disclosing the humidity cross-response to H_2_S sensing. Tests confirmed that humidity decreases the baseline resistance, hampers the H_2_S sensor’s signal (i.e., relative response (RR) = *R*_a_/*R*_g_), and increases the limit of detection (LOD). At 1 ppm, the H_2_S sensor’s signal decreases from an RR of 2.4 ± 0.1 at 0% RH to 1.9 ± 0.1 at 80% RH, while the LOD increases from 210 to 380 ppb. Utilizing a suitable thermal treatment, here, we report an amorphization procedure that can be easily extended to a large variety of TMDs and MCs, opening extraordinary applications for 2D layered amorphous metal oxide gas sensors.

Two-dimensional (2D) layered transition metal dichalcogenide (TMD) and metal chalcogenide (MC) semiconductors, with near atomic-scale thickness, have been extensively proposed in the past decade as alternative materials for traditional nanocrystalline metal oxides (MO) for gas sensing applications.^1−4^ Key advantages of these interfaces are represented by their high surface-to-volume ratios,^5^ the direct-to-indirect band gap transition,^6,7^ the occurrence of chemical terminations like edges, boundaries, and surface vacancies,^8−10^ and the engineered functionalities by metal nanoparticle decoration or substitutional doping.^11,12^ Despite these features, a substantial disadvantage of TMDs and MCs, adversely affecting sensors’ signal reproducibility, is represented by their intrinsic thermodynamic instability (Δ*G* < 0), leading to spontaneous oxidation in dry-/wet-air laboratory conditions.^13,14^ In details, the displacement of sulfur, selenium, and tellurium atoms, operated by ambient O_2_ in MoS_2_ and WS_2_ sulfides,^15,16^ MoSe_2_, WSe_2_, InSe, GaSe, and SnSe_2_ selenides,^17−20^ and MoTe_2_ and WTe_2_^21,22^ tellurides, stimulates the nucleation over step edges of amorphous oxidized states, which proceeds through basal planes, eventually passivating all the flake’s surface. This phenomenon is further enhanced when the sensor’s operating temperature (OT) is increased in the range of 25–150 °C to compensate for irreversible adsorption of gas molecules, as frequently experienced in metal oxide and 2D layered sensors.^23,24^

Spontaneous oxidation of chalcogenides represents, indeed, an excellent opportunity to synthesize new kinds of interfaces comprising thin layers of amorphous metal oxides grown over 2D layered crystalline materials, yielding *a*-MO/TMD and *a*-MO/MC heterostructures with unexpected applications in the field of catalysis and gas sensing.^25^ On this account, we recently demonstrated by means of experiments and theory that it is possible to synthesize *a*-SnO_2_/SnSe_2_ heterostructures to detect NO_2_, H_2_, NH_3_, and humidity^26,27^ by controlled oxidation in air of 2D exfoliated SnSe_2_ layers. Besides their excellent gas sensing response, these 2D amorphous/2D crystalline heterostructures unfortunately retain some remarkable limitations. The difficulty of controlling the final thickness of the growing *a*-SnO_2_ oxide over the 2D crystalline platform and the risk that the *a*-SnO_2_ film is not self-passivating, i.e., not protecting the underlying 2D layer from further oxidation, make their practical exploitation challenging.^28^

Departing from liquid-phase exfoliated SnSe_2_ layers (10–30 nm thick) and controlling the oxidation in air at 250 °C for two weeks, at temperatures below the crystallization temperature of *a*-SnO_2_ oxide (i.e., ≈280 °C), we show for the first time that the oxidation process of 2D SnSe_2_ can be successfully driven to the “core” of the flakes, yielding single-phase, fully amorphous 2D *a*-SnO_2_, which is stable up to 250 °C and sensitive to H_2_S gas (400 ppb to 1.5 ppm) and to humid air (10–80% RH (relative humidity) (RH @ 25 °C)) at a 100 °C operating temperature. Layered amorphous metal oxide sensors (LAMOS) like *a*-SnO_2_ can be easily manufactured in a thin-film form by standard spin-coating deposition techniques representing a new interface for chemoresistive gas sensing applications. The amorphization thermal oxidation process here validated can be extended to a large variety of TMDs and MCs, opening new opportunities for “LAMOS” interfaces with unexplored surface-science capabilities, probably well beyond gas sensing applications.

## Results and Discussion

**Figure 1:**
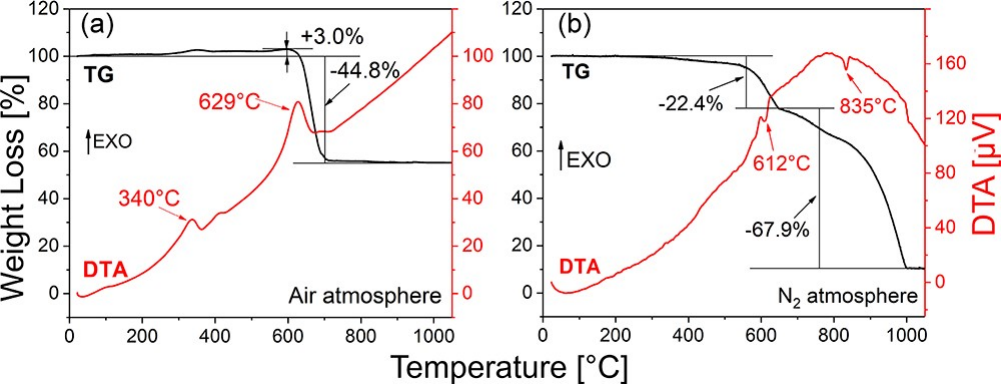
Thermogravimetric (TG) and differential thermal analysis (DTA) plots of as-exfoliated SnSe_2_ flakes heated in air (a) and nitrogen (b) atmospheres at 5 °C/min from 25 to 1050 °C. Black and red lines refer to TG and DTA signals, respectively.

Thermal stability of exfoliated SnSe_2_ flakes has been preliminarily investigated by simultaneous thermogravimetric (TG) and differential thermal analysis (DTA) techniques in air and nitrogen atmospheres by heating as-exfoliated SnSe_2_ at 5 °C/min, to maximize the gain of the TG signal, to 1050 °C as shown in Figure 1a,b. In the range of 200–600 °C (Figure 1a), a weight increase of approximately 3.0% is recorded in static air corresponding to the onset of an exothermic peak of the DTA signal located at 340 °C. This result (i) is congruent with previous theoretical investigations predicting the formation of an intermediate SnSe_2_O_2_ oxide^26,29^ and (ii) rules out any sublimation of SeO_2_ species (eventually associated with a weight loss in Figure 1a), as previously found for SnSe_2_ powders.^30^ In the temperature range of 600–800 °C, the measured −44.8 ± 0.7% weight loss in air well agrees with the theoretical weight loss of −45.5% corresponding to the complete oxidation of SnSe_2_ to SnO_2_ (maximum rate at 629 °C).

Heating in a N_2_ atmosphere in the 200–1000 °C range, as shown in Figure 1b, inhibits any weight gain, confirming the absence of substantial oxidation phenomena in a nitrogen atmosphere with increasing temperature. The measured weight losses in N_2_ of −22.4% (at 612 °C) and −67.9% (at 835 °C) can be further attributed to the conversion of SnSe_2_ to Sn_2_Se_3_^31,32^ and the complete removal of Se and partial sublimation of Sn atoms, respectively,^31^ as reported in the literature.

**Figure 2:**
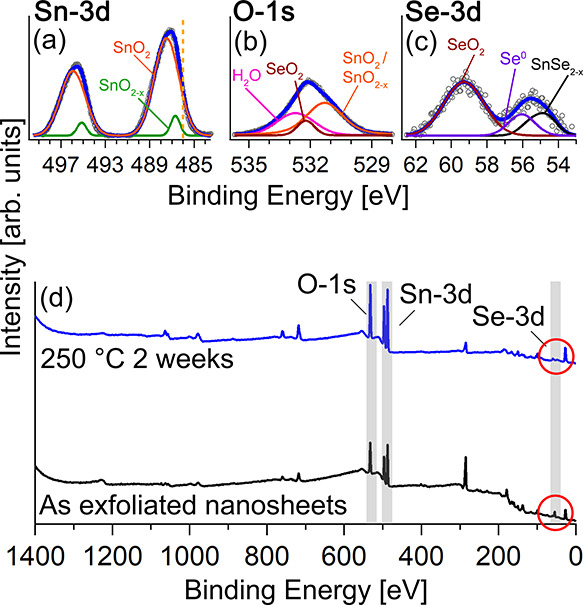
Deconvoluted Sn 3d (a), O 1s (b), and Se 3d (c) core-level spectra of SnSe_2_ flakes after two weeks of annealing at 250 °C. Raw data (empty gray circles) and cumulative fits (blue lines) are reported. (d) Survey XPS spectra of SnSe_2_ exfoliated nanosheets measured before (black) and after annealing (blue). Shaded gray areas highlight O 1s, Sn 3d, and Se 3d core levels.

The surface chemical composition of annealed SnSe_2_ at 250 °C for two weeks has been investigated by XPS analysis. Figure 2a–c shows the detailed XPS Sn 3d, O 1s (b), and Se 3d (c) core-level spectra of SnSe_2_ flakes after two weeks of annealing in static air at 250 °C. Deconvolution of the Sn 3d_5/2_ core-level spectrum (Figure 2a) and quantitative analysis of the phases’ composition shown in Table 1 highlight the complete oxidation of the surface, as demonstrated by the occurrence of two contributions ascribed to (i) stoichiometric SnO_2_ (orange line) with maximum peak intensity of the *j* = 5/2 component centered at 487.4 eV^33^ covering approximately 97% of the whole spectral area and (ii) defective SnO_2–*x*_ (green line) at 486.6 eV^34^ representing nearly 3% of the entire signal.

**Table 1:** Relative and Cumulative Surface Atomic Concentrations (at. %) of Sn, O, and Se Elements

	Sn 3d_5/2_	O 1s	Se 3d_5/2_
single spectral area [arbitrary units]	1417	6143	245
relative percentages (with respect to cumulative counts of Sn 3d, O 1s, and Se 3d)	18%	79%	3%

	SnO_2_	SnO_2–*x*_	H_2_O	SeO_2_	SnO_2_	SeO_2_	Se(0)	SnSe_2–*x*_
BE [eV]	487.5	486.6	532.7	532.2	531.3	59.3	56	54.6
area [arbitrary units]	5961	168	1733	457	2148	109	29	36
relative %	97%	3%	40%	10%	50%	63%	17%	21%
cumulative %	18%	1%	31%	8%	38%	2%	1%	1%

The lack of any signal at 486.1 eV,^29,35^ corresponding to the vertical dashed line of Figure 2a, confirms the absence of Sn–Se chemical bonds in the annealed sample. These results are congruent with O 1s and Se 3d core-level spectra of Figure 2b,c respectively. Specifically, the O 1s spectrum can be decomposed into three signals. The main peak, representing ≈50% of the total spectral area, is centered at 531.3 eV corresponding to Sn–O.^36−38^ Those located at 532.2 and 532.7 eV are ascribed to SeO_2_^39,40^ (≈10%) and adsorbed H_2_O^41^ (≈40%), respectively. Finally, the Se 3d core-level spectrum (Figure 2c) comprises three contributions at 59.3, 56, and 54.6 eV, associated to SeO_2_,^41^ metallic Se,^42^ and SnSe_2–*x*_,^18^ respectively.

According to Table 1, having set at 100% the cumulative spectral areas of Sn 3d, O 1s, and Se 3d, net of the elemental sensitivity of the XPS technique,^43^ the relative atomic percentages of Se, Sn, and O yield Se 3d ≈ 3%, Sn 3d ≈ 18%, and O 1s ≈ 79%. In particular, the elemental ratio of O:Sn is found to be close to 2:1, which supports the occurrence of the SnO_2_ phase.

Notably, the negligible contribution of the Se 3d signal in the annealed sample is confirmed in the survey spectra of Figure 2d exhibiting (compared by electronic magnification of the spectra in the red circles) the vanishing of the Se 3d signal in the annealed sample (blue line) with respect to the exfoliated SnSe_2_ one (black line). It may be concluded that after controlled thermal treatment, the gas-responding surface of the annealed SnSe_2_ flakes comprises almost stoichiometric SnO_2_ and a negligible amount, approximately close to the instrumental resolution of the XPS equipment (±1%), of SeO_2_ phases.

**Figure 3:**
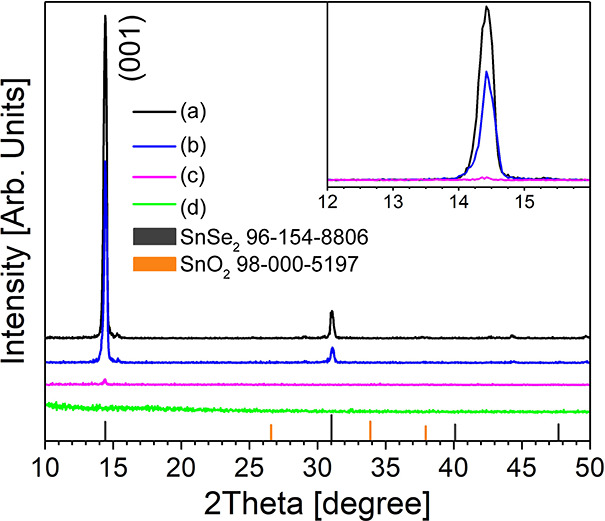
Grazing incidence (GI)-XRD diffraction patterns of as-exfoliated SnSe_2_ flakes (a) and SnSe_2_ annealed at 250 °C for one week (b), 250 °C for two weeks (c), and 250 °C for two weeks + one extra week at 280 °C (d).

The amorphization process of SnSe_2_ at different annealing times and temperatures has been investigated by the grazing incidence (GI)-XRD technique over spin-coated and annealed thin films deposited over silicon substrates. According to Figure 3, as-exfoliated SnSe_2_ exhibits a major diffraction peak (black line) corresponding to the (001) plane of SnSe_2_ at 2θ = 14.4° (ICDD card no. 96-154-8806). At 250 °C, with proceeding the annealing time from one week (blue line) to two weeks (magenta line), the (001) peaks almost disappear (see also the inset of Figure 3), confirming the effectiveness of the amorphization process. The two-weeks annealed sample at 250 °C (magenta), further annealed for an extra week at 280 °C, retains its amorphous structure (green), highlighting no substantial recrystallization phenomena of amorphous *a*-SnO_2_ into crystalline SnO_2_ as it will be further discussed in the HRTEM characterization.

**Figure 4:**
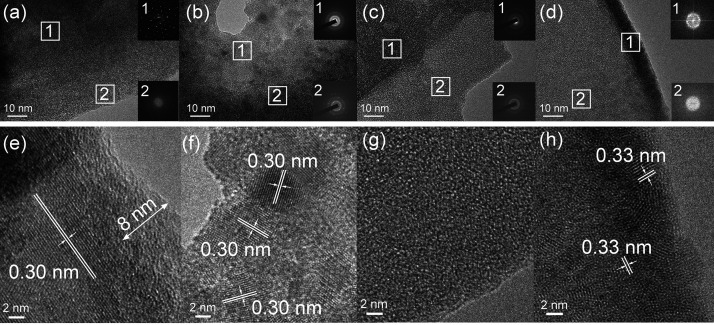
HRTEM pictures of (a,e) as-exfoliated SnSe_2_ flakes and SnSe_2_ (b,f) annealed at 250 °C for one week, (c,g) annealed at 250 °C for two weeks, and (d,h) annealed at 250 °C for two weeks + one extra week at 280 °C. SAED patterns corresponding to the identified regions are shown in the insets.

The microstructural evolution of SnSe_2_ flakes annealed at different times/temperatures, over selected flake’s regions (Figure S1), has been characterized by HRTEM microscopy and is shown in Figure 4. As-exfoliated SnSe_2_ (Figure 4a) exhibits a fully crystalline structure (SAED1), corresponding to the inner region of the flakes, with an interplanar distance of 0.30 nm (Figure 4e), congruent with the (001) crystallographic plane of SnSe_2_ determined by GI-XRD. Notably, an amorphous edge (SAED2), extending by approximately 8 nm inside the flake’s terrace, is also detectable (Figure 4e), which is congruent with an oxidation process mechanism advancing from the outside to the inside of the flakes, as extensively reported for TMD and MD materials.^13,18^ Despite our previous research demonstrating no significant edge oxidation phenomena of liquid-phase exfoliated SnSe_2_,^28^ in this case, we attribute the step-edge amorphization process of SnSe_2_ to the combined action of different sonicating conditions and the use of a different solvent (here NMP). With annealing at 250 °C for one week (Figure 4b,f), the degree of crystallization decreases compared to the as-exfoliated sample, as confirmed by the formation of halos in SAED patterns (i.e., compare SAED1 of Figure 4a with SAED2 of Figure 4b). The onset of an amorphization phenomenon is confirmed in Figure 4f where a patchwork of crystalline/amorphous phases is clearly displayed. With annealing for two weeks at 250 °C, crystalline domains of the parent SnSe_2_ completely disappear, as exhibited in Figure 4c,g, confirming the completeness of the amorphization process. To conclude, we also tried to investigate the recrystallization mechanism of amorphous *a*-SnO_2_ into crystalline SnO_2_, as shown in Figure 4d,h. We found that by an extra week of annealing at 280 °C, crystalline domains are initially formed on step edges, as shown in Figure 4d with corresponding interatomic plane distances of 0.33 nm (Figure 4h), attributed to the (110) plane distances of tetragonal rutile SnO_2_.^44^ It turns out that the recrystallization of *a*-SnO_2_ into crystalline SnO_2_ proceeds from the outside to the inside of the flake, considering that no nucleating SnO_2_ crystallites are visible inside the flakes as shown in Figure 4d,h. The limited extension of the crystalline domains with respect to the amorphous ones shown in Figure 4d,h may also explain the lack of any diffraction peak attributed to crystalline SnO_2_ in the GI-XRD pattern of Figure 3.

In conclusion, the whole amorphization process here presented, comprising the recrystallization of *a*-SnO_2_ to SnO_2_, possibly represents interesting evidence of a nearly topotactic transformation of a 2D SnSe_2_ metal chalcogenide into a 2D *a*-SnO_2_ metal oxide, which includes loss of selenium and oxygen gain so that the final *a*-2D structure, retains the same bidimensional feature of the original material. This process represents a matter worthy of further investigation, eventually constituting a promising route to synthesize new metal oxide *a*-2D interfaces for gas sensing applications. Combining XPS, XRD, and HRTEM observations, it may be concluded that by annealing in air for two weeks at 250 °C, approximately 30 °C below the onset of the recrystallization temperature of *a*-SnO_2_, the complete oxidation/amorphization of exfoliated 2D SnSe_2_ flakes into 2D *a*-SnO_2_ is achieved.

### Gas Sensing Response to H_2_S and Humidity

**Figure 5:**
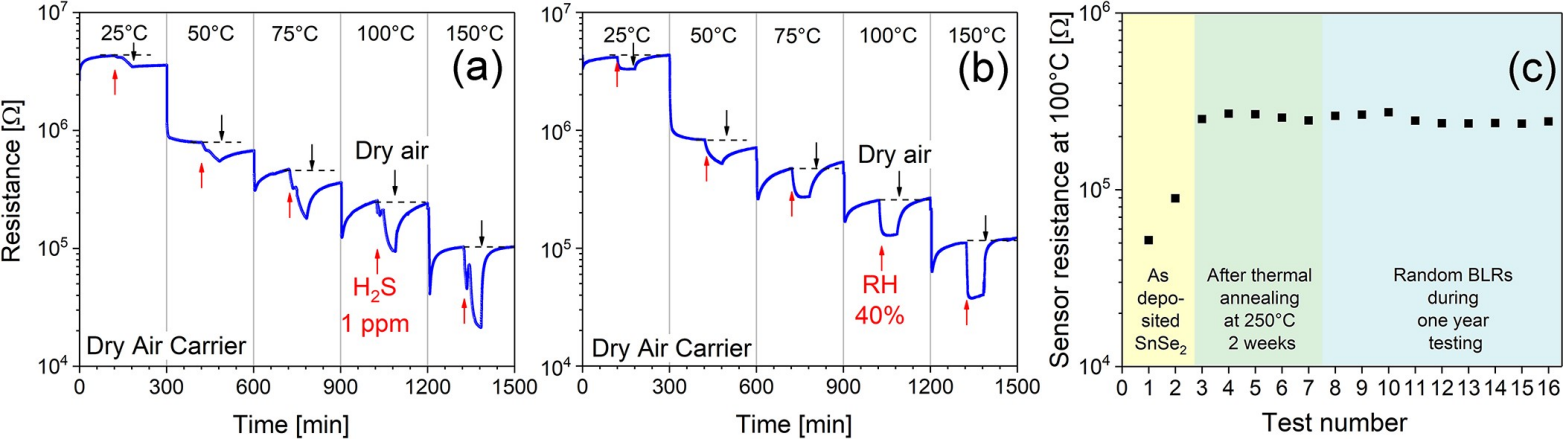
Electrical responses of the two-weeks/250 °C annealed *a*-SnO_2_ sensor in dry air at different OTs (from 25 to 150 °C) to (a) 1 ppm H_2_S and (b) 40% RH (RH @ 25 °C). (c) Baseline resistance evolution in dry air at a 100 °C OT of (i) as-exfoliated SnSe_2_ (yellow region) and (ii) as-annealed (two weeks/250 °C) *a*-SnO_2_ (green region) and (iii) *a*-SnO_2_ thin-film baseline resistance randomly measured during one-year conditioning at 100 °C (blue region).

The best operating temperature (OT) for H_2_S and H_2_O of the two-weeks/250 °C annealed *a*-SnO_2_ sample has been identified in light of two main features of the sensor’s response: (i) sensor’s signal amplitude, as represented by the relative response ratio (RR = *R*_air_/*R*_gas_), and (ii) recovery of the baseline resistance after gas desorption (BLR, i.e., the resistance in air at equilibrium). Tests have been carried out in the OT range of 25–150 °C in dry-air carrier gas exposing the film to 1 ppm H_2_S and 40% relative humidity (40% @ 25 °C), as shown in Figure 5a,b respectively. By increasing the operating temperature, the sensor shows a monotonic decrease in the BLR, indicating a semiconducting behavior with an *n*-type response to both H_2_S and humid-air reducing gases, consistent with preliminary results on *a*-SnO_2_/SnSe_2_ interfaces^28^ and metal oxide SnO_2_ sensors.^45^

According to Figure 5a, in the temperature range of 25–75 °C, the sensor displays a low signal response to 1 ppm H_2_S and no recovery of the BLR (dashed black lines in the figure) even after 2 h of dry-air purge. With increasing the OTs, both the sensor’s signal and desorption kinetics improve. The BLR is fully recovered, departing from the 100 °C OT, topping the best sensor’s signal response at 150 °C. Poor recovery of the BLR in the temperature range of 25–75 °C, shown in Figure 5a, is a typical feature of a large variety of 2D TMD/MC gas sensor interfaces operated at near-room temperatures.^46^ On this account, to improve gas desorption rates and BLR recovery, UV–vis light irradiation or increasing the sensor’s operating temperature in the 75–150 °C range has been proposed for both traditional metal oxide^47,48^ and 2D TMD/MD sensors.^49,50^ These strategies indeed show remarkable limitations. UV-light irradiation of 2D TMDs/MDs requires almost monolayer thin interfaces (i.e., <5–7 nm),^46,51,52^ whereas thermal heating at higher temperatures stimulates fast surface oxidation, hampering BLR reproducibility over the long run. On this account, *a*-SnO_2_ annealed at 250 °C can be safely operated in the temperature range of 100–150 °C without any risk of further degradation or recrystallization, providing an effective solution for fast BLR recovery and improved sensor’s signal reproducibility. Water vapor at 40% RH (RH @ 25 °C) behaves like H_2_S, as shown in Figure 5b, with improved sensor’s signal and BLR recovery in the temperature range of 100–150 °C (see also dynamic humidity responses in the range of 25–75 °C in Figure S4). Notably, a complete recovery of the baseline is also recorded at 25 °C, possibly on account of a protonic (H^+^) Grotthuss chain-like conduction mechanism induced by physisorbed water at lower temperatures.^53^ Departing from the 100 °C OT, most of the physisorbed water is removed^54^ and water vapor responds as a reducing gas as previously reported.^55^

Baseline resistance reproducibility over the long run, under sustained OTs and dry/wet conditions, also represents a key issue for the exploitation of this new kind of interface. Figure 5c shows the evolution of the BLR in dry air recorded at a 100 °C OT of (i) as-exfoliated SnSe_2_ (yellow region), (ii) two-weeks/250 °C annealed SnSe_2_ (green region), and (iii) *a*-SnO_2_ thin films after one-year conditioning at 100 °C (blue region). The amorphization process, producing truly stable *a*-SnO_2_ oxide, sharply increases the BLR in dry air (yellow-green region), which finally stabilizes, exhibiting excellent reproducibility and stability (±5% of the BLR variation) over the long run (blue region). One-year recordings of the electrical resistance of *a*-SnO_2_ to 1 ppm H_2_S gas and 40% humidity, shown in Figure S5, confirmed a remarkable reproducibility with an associated uncertainty of the sensor’s signal to H_2_S gas and humidity as low as ±0.1 and ±0.2, respectively.

**Figure 6:**
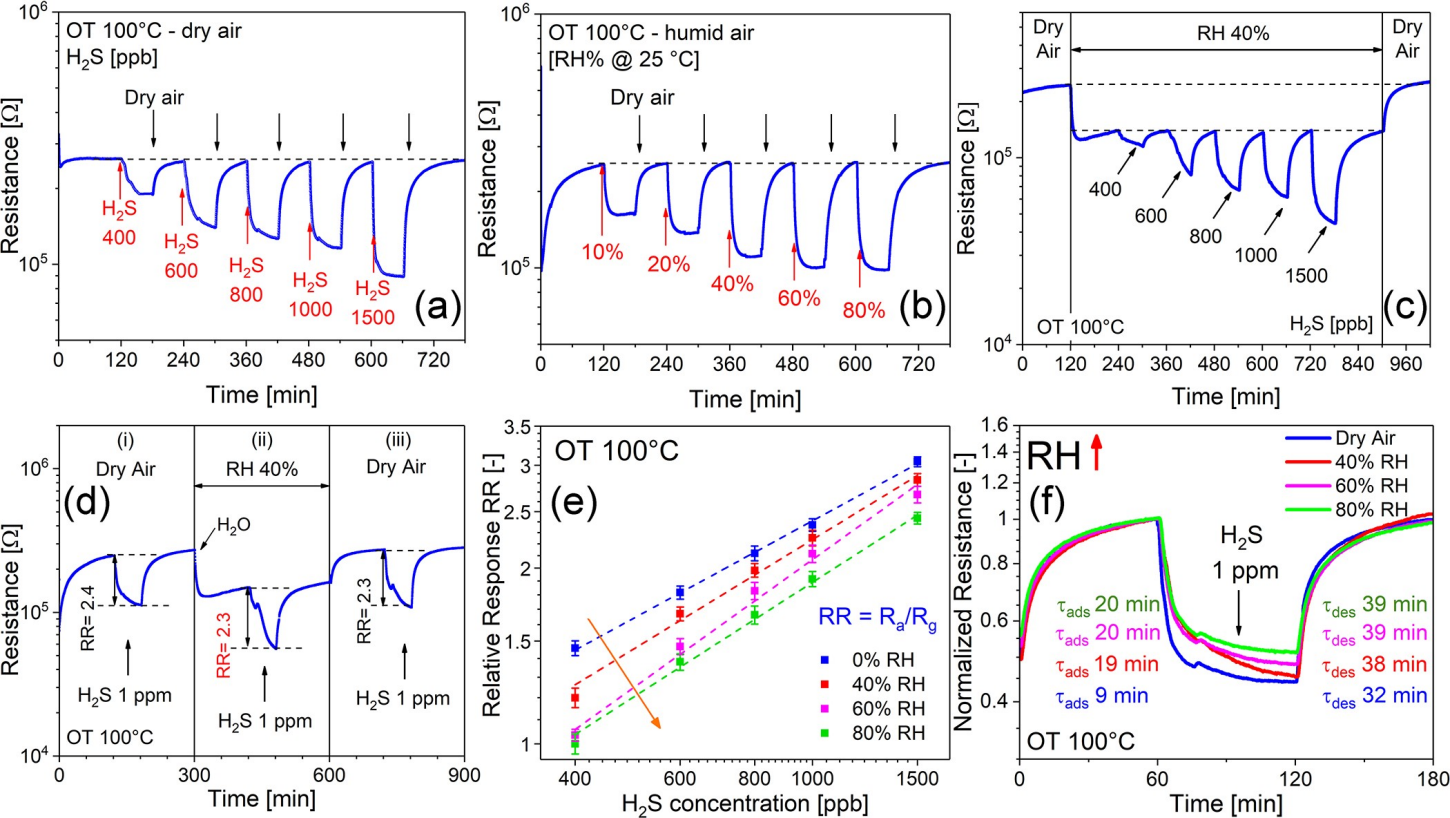
Dynamic electrical responses in dry air at a 100 °C OT to (a) H_2_S (400 ppb to 1.5 ppm) and (b) H_2_O in the range of 10–80% RH (RH @ 25 °C). (c) Dynamic electrical responses of *a*-SnO_2_ at a 40% RH background and increasing concentrations of H_2_S (400 ppb to 1.5 ppm). (d) 40% RH humidity cross-response to 1 ppm H_2_S at a 100 °C OT: (i) first step in dry air and 1 ppm H_2_S; (ii) second step at a 40% RH background and 1 ppm H_2_S; (iii) third step, equivalent to (i), to check for short-term repeatability. (e) Log/log calibration plots at different RH values from 0 to 80% (as to the arrow) to increasing concentrations of H_2_S, measured at a 100 °C OT (associated standard deviations calculated over a set of five consecutive measurements); (f) adsorption and desorption times of H_2_S (1 ppm) to increasing RH values as to the red arrow.

Figure 6a,b shows the dynamic resistance changes at a 100 °C OT of *a*-SnO_2_ to H_2_S (400 ppb to 1.5 ppm range) and H_2_O (10–80% RH range, RH @ 25 °C) in dry-air carrier gas, respectively. Both H_2_S and H_2_O at a 100 °C OT yield strong interactions with the *a*-SnO_2_ surface, indeed with excellent recovery of the BLR following each step of gas/humidity purge. The effect of the 40% RH background to the dynamic H_2_S response shown in Figure 6c (see also Figure S6 at 60 and 80% RH backgrounds) highlights that humid water decreases the BLR, though preserving a satisfactory H_2_S gas dynamic modulation.

The assessment of the sensor’s signal variations when both humidity and H_2_S compete at the same time over the sensor’s surface represents another key issue of the sensor’s performance. This feature, known as humidity cross-response (CR) to H_2_S sensing, is shown in Figure 6d. The test comprises (i) a first step in dry air and 1 ppm H_2_S, (ii) a second step at a 40% RH background and 1 ppm H_2_S, and (iii) a third step, equivalent to (i), to check for short-term repeatability. Comparing the sensors’ signals in dry (i) and wet conditions (ii), no relevant changes of the RRs (RR = *R*_a_/*R*_g_) are displayed, considering that 1 ppm H_2_S yields an RR of 2.4 ± 0.1 in dry air and an RR of 2.3 ± 0.1 in humid air (40% RH), provided an associated sensor’s signal uncertainty of ±0.1 (measured over a set of five consecutive measurements). Moreover, no substantial changes in the electrical response are displayed comparing panels (i) and (iii), demonstrating an excellent short-term repeatability.

The humidity cross-response (CR) to H_2_S sensing in the whole gas/humidity concentration range at a 100 °C OT, as represented by the log–log calibration plots of the sensor’s signal (i.e., RR = *R*_a_/*R*_g_) vs H_2_S gas concentrations and different RH values, is shown in Figure 6e. Increasing relative humidity from dry conditions to 80% RH (as to the direction of the arrow), sensor’s signal amplitude decreases. Specifically, at 1 ppm H_2_S, sensor’s signal amplitude decreases from an RR of 2.4 ± 0.1 at 0% RH to an RR of 1.9 ± 0.1 at 80% RH. Moreover, congruent with this tendency, water vapor has a negative effect on increasing the H_2_S limit of detection (LOD). By numerical extrapolation of the calibration lines of Figure 6e (according to the methods in Supporting Information, Section S3), the theoretical LOD increases from 210 ppb at 0% RH to 380 ppb at 80% RH, confirming the inhibiting effect of water vapor upon H_2_S sensing. The antisynergistic interaction of water vapor upon H_2_S detection is also confirmed in Figure 6f and Figure S7, showing the adsorption/desorption times of 1 ppm H_2_S as a function of the humidity content. Increasing RH% (as to the direction of the red arrow), the adsorption time of 1 ppm H_2_S increases from 9 (@ 0% RH) to 20 min (@ 80% RH), while the sensors’ signal amplitude decreases from an RR of 2.4 ± 0.1 at 0% RH to an RR of 1.9 ± 0.1 at 80% RH. As a concluding remark, the adsorption–desorption times here reported (order of minutes) are in most cases much longer than those frequently reported in the literature (order of seconds). On this account, it should be noted that response times are mostly dependent on the experimental conditions like the humidity content and gas fluid dynamics inside the test cell. As to the latter, the theoretical residence time of the gas (TRT) given by the ratio between the cell volume [cm^3^] and the gas flow rate [cm^3^/min] may significantly differ from the mean residence time (MRT), representing the actual time to completely fill/empty the test cell. In a previous paper utilizing NO_2_ as a marker gas, we found that the characteristic MRT of our experimental setup is between 4 and 5 min^28^ with respect to a TRT of only 1 min. Humidity as well influences response time. As shown in Figure 6f, the response time to 1 ppm H_2_S almost doubles from dry to 80% RH wet conditions. In conclusion, when comparing response times of different sensors/gases, the experimental setup, the humidity content, and operating temperatures should always be considered and eventually normalized.

Gas sensing relative responses (RR = *R*_a_/*R*_g_) of selected sensors, calculated by normalizing literature data to 1 ppm H_2_S in dry air and at different OTs, are compared in Table 2. In addition to traditional porous metal oxide sensors^56^ and metal oxide heterostructures, which guarantee the most favorable catalytic efficiency, *a*-SnO_2_ performs better than traditional 2D TMD/MD^1−4^ sensors operated in the same temperature range (150–200 °C). 2D *n*-*n*/*p*-*n* heterostructures^1−4^ operating at room temperature show RRs slightly smaller than that of *a*-SnO_2_, excluding *n*-*n* SnSe_2_/SnO_2_^29^ with an associated sensor’s signal as high as 7.5 to 1 ppm H_2_S gas. Regarding *n*-*n*/*p*-*n* 2D heterostructures, which can be classified as “decorated” interfaces since they almost comprise 3D crystalline metal oxide nanoparticles grown over 2D TMD/MD flakes, it is not usually appreciated that the naked 2D TMD/MD surfaces of the heterostructure, i.e., the ones not protected by the “decoration”, are likely to be oxidized in dry/wet air in the long run, negatively affecting the reproducibility of the electrical signal.

**Table 2:** Comparison of the H_2_S Gas Sensing Performances of Different Sensors’ Interfaces Obtained by Normalizing Literature Data to 1 ppm H_2_S in Dry Air

sensing materials	H_2_S [ppm]	response *R*_a_/*R*_g_ [−]	OT [°C]	ref.
3D metal oxide^56^				
SnO_2_ porous NF	1	14.3	350	(45)
ZnO (thin film)	1	4.3	330	(57)
WO_3_	1	4.5	330	(58)
CuO	1	1.8	135	(59)
p-Co_3_O_4_	1	2.0	210	(60)
3D metal oxide heterostructures				
MoO_3_/SnO_2_	1	9	115	(61)
Cu_2_O/CuO	1	6.3	95	(62)
rGO/WO_3_	1	7.7	330	(58)
ZnO/CuO	1	6.7	25	(63)
PdRh ZnO-HC	1	2.9	260	(64)
MoO_3_/WO_3_	1	14.0	250	(65)
2D transition metal dichalcogenides (TMDs) and metal chalcogenides (MCs)^1−4^				
p-type WS_2_	1	1.1	200	(66)
n-type WS_2_	1	1.2	150	(67)
MoSe_2_	1	1.2	200	(68)
SnSe_2_	1	1.8	200	(29)
2D *n*-*n*/*p*-*n* heterostructures^1,2,4^				
SnO_2_/SnSe_2_	1	7.5	25	(29)
CuO/MoS_2_	1	1.1	25	(69)
Ag-MoSe_2_/rGO	1	1.2	25	(70)
SnSe_2_/WO_3_	1	1.3	25	(71)
2D LAMOS (this work)				
*a*-SnO_2_	1	2.4	100	this work

### Theoretical Model of H_2_S and Humidity Adsorption

**Figure 7:**
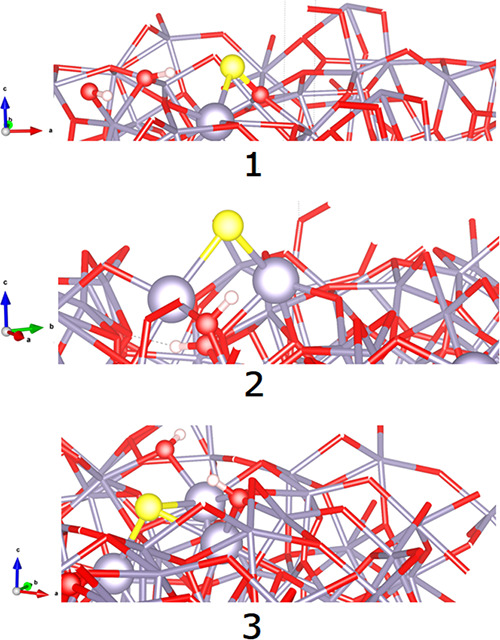
The three most stable optimized structures of H_2_S anchored on top of the *a*-SnO_2_ as obtained by DFT calculations on top of AIMD-calculated trajectories (see Computational Methods in Supporting Information, Section S4 for details) (yellow, S; mauve, Sn; red, oxygen; white, H atoms).

To support the experimental results, we carried out DFT atomistic simulations of H_2_S molecule adsorption utilizing the same theoretical model of water molecules anchoring on the amorphous *a*-SnO_2_/SnSe_2_ nanosheet (NS).^28,72,73^ Three stable geometries, all chemisorbed, whose structure and energetics are shown in Figure 7 (1–3) and Table 3, respectively, are found according to the theoretical procedure described in the Supporting Information. Specifically, Figure 7 exhibits three configurations with broken H–S bonds (two O–H bonds are similarly formed), supporting previous findings over crystalline SnO_2_ that molecularly adsorbed H_2_S attack is not favored.^74^ The first structure (**1**, see Figure 7, top) is characterized by a S atom bound both to one Sn (*d*_Sn–S_ = 2.47 Å) and to one O atom (*d*_Sn–O_ = 1.68 Å). This mechanism is exothermic by 1.38 eV (following eq S1 in the SI). Bader analysis confirms what is expected on the presence of a newly formed S–O bond, that is, a slightly positively charged sulfur atom (+0.19) because of the larger electronegativity of oxygen.

**Table 3:** Main Structural and Thermodynamic Data of the Three Most Stable Anchoring Mechanisms^a^

			Bader charge [charge units]		
adsorbate	structure	*E*_ads_ [eV]	S	H(1)	H(2)
H_2_S	**1**	–1.38 (−0.28)	+0.19	+0.58	+0.60
H_2_S	**2**	–4.52 (−3.42)	–0.71	+0.60	+0.64
H_2_S	**3**	–3.65 (−2.55)	–0.72	+0.60	+0.59

			O	H(1)	H(2)
H_2_O	**C2**	–1.24 (−2.02)	–0.52	–0.09	+0.15

a See Figure 7, **1**–**3**. *E*_ads_ is in eV, and the Bader charge is in elementary charge units. *E*_ads_ are values obtained by combining AIMD+DFT approaches for *E*_surf_H2S_ and (in brackets) values obtained still by combining AIMD+DFT approaches for the calculation of both *E*_surf_H2S_ and *E*_surf_ terms in eq S1 in Supporting Information, Section S4, which is the same for **C2**, i.e., the most stable anchoring mechanism of H_2_O as described in ref (28). A Bader charge of >0 indicates charge transfer from the adsorbed species to the *a*-SnO_2_ surface. A Bader charge of <0 indicates electron charge transfer from the *a*-SnO_2_ surface to the adsorbed species.

The second structure (**2** in Figure 7, middle) is the most thermodynamically stable (*E*_ads_ = −4.52 eV) and is characterized by a sulfur atom bound to two Sn atoms. Such two Sn–S bonds are 2.44 and 2.46 Å, respectively, showing that the enhanced stability may be correlated with the shorter Sn–S bond length. In this case, the Bader analysis reveals an enhanced electron localization on S (−0.71), a fingerprint of the more marked electronegativity of S (compared to that of Sn, i.e., 2.58 vs 1.96).^75^ The last structure (**3** in Figure 7, bottom), where the S atom is three-fold coordinated with Sn atoms (*d*_Sn–S_ = 2.46, 2.55, and 2.94 Å), is still markedly stabilized, compared to the reactants, with an adsorption energy of −3.65 eV and charge distribution on S (−0.72) very close to that in **2**. Regarding the H(1) and H(2) hydrogen adsorption modes, deriving from the rupture of the H_2_S molecule, the Bader charge associated to the formation of O–H bonds with the O atoms at the surface is similar for all the three structures with a positive sign confirming the direction of the charge transfer from the adsorbed species to the *a*-SnO_2_ surface.

**Figure 8:**
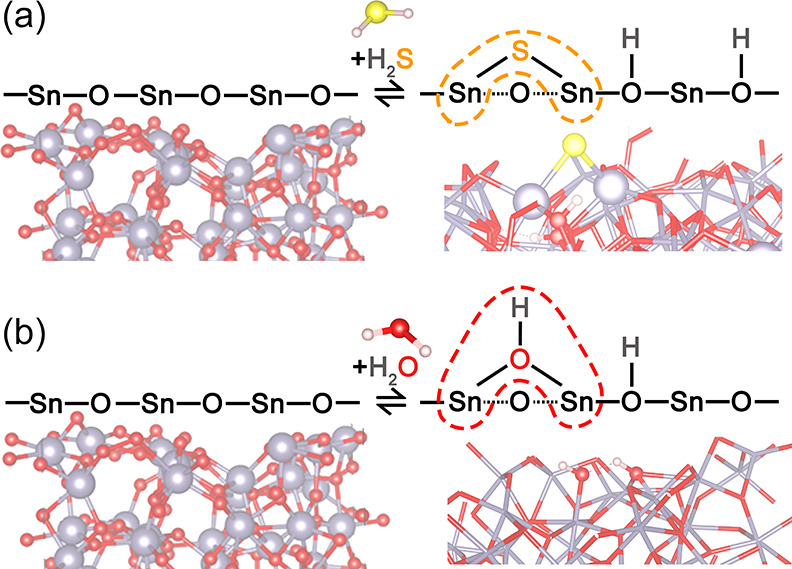
Schematization of the adsorption attack of H_2_S and H_2_O over the optimized clean *a*-SnO_2_ surface. The left-hand side refers to the optimized initial clean *a*-SnO_2_ system, and the right-hand side refers to the adsorption modes of H_2_S (a) and H_2_O (b). Yellow, S; mauve, Sn; red, oxygen; white, H atoms.

An overall schematization of the most stable H_2_S adsorption configuration over the optimized initial clean *a*-SnO_2_ surface shown on the right-hand side of Figure 8a confirms the occurrence of a homolytic dissociation of H_2_S with the formation of a rooted S atom, two-fold coordinated with Sn lattice atoms indicated as (S)_2Sn_, and two rooted hydroxyls group (i.e., (OH)_O_).

In the same fashion, as shown in the right-hand side of Figure 8b following a heterolytic rupture of OH bonds in H_2_O,^28^ water vapor chemisorbs over the *a*-SnO_2_ surface with the formation of a rooted hydroxyl, two-fold coordinated with surface Sn atoms indicated as (OH)_2Sn_, and one rooted hydroxyl group (OH)_O_. Corresponding energetics and Bader charges are shown in the last line of Table 3, referring to the most stable H_2_O attack (specifically **C2** in ref (28)).

### Gas Sensing Mechanism

At a 100 °C operating temperature, the adsorption of H_2_S gas and H_2_O over *a*-SnO_2_ is experimentally transduced by a decrease in the sensor’s resistance, as displayed in Figure 6a,b. In our case, we consider a clean *a*-SnO_2_ amorphous surface where both H_2_S and H_2_O compete, as depicted in Figure 8a,b, at the same time over the same *a*-SnO_2_ adsorption sites, according to the following reactions:
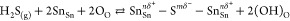
1
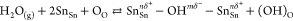
2
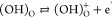
3

Compliant to theoretical computations, the positive (+) overall Bader charge balance of H_2_S adsorption (see structure **2**, Table 3), derived from the balance of *n*δ^+^, *m*δ^–^, and hydroxyl–hydrogen atomic charge distribution as schematized by reaction 1, provides an electron enrichment of the *a*-SnO_2_ surface, which is congruent to the decrease in the sensor’s resistance with H_2_S gas as shown in Figure 6a. Conversely, according to reaction 2, the negative (−) overall Bader charge balance of H_2_O adsorption (see structure **C2**, Table 3(28)) confirms the electron depletion of the surface, which conflicts with the decrease in the sensor’s resistance recorded in Figure 6b. This apparent contradiction can be explained accounting for the lower electron affinity of the rooted hydroxyls (OH)_0_,which are easily ionized according to reaction 3, providing extra electrons (e′) that outweigh the negative charge depletion operated by water adsorption.

Fundamental investigations on the temperature interaction of water vapor over crystalline SnO_2_ utilizing operando DRIFT spectroscopy^76^ confirmed, opposite to our dissociative adsorption mechanism, that neither physisorption nor dissociative water adsorption occurs over the surface of crystalline SnO_2_ in the temperature range of 100–150 °C. On this account, theoretical vs experimental conditions should always be considered. Our theoretical approach refers to a clean *a*-SnO_2_ amorphous interface, while DRIFT experiments specifically apply to sol–gel-prepared crystalline SnO_2_. Overall, it may be concluded that regardless of (i) the preparation conditions, (ii) the crystalline or amorphous nature of the interface, and (iii) the 2D or 3D geometry of the platform, in all cases, humidity substantially affects the resistance of the device. Specifically, it seems that the 2D layered nature of TMDs and MDs does not gain substantial advantages to improve humidity cross-interference compared to traditional metal oxide sensors. However, theoretical and practical investigations of the water vapor adsorption mechanism over 2D layered materials are still young, while effective practical strategies to promote selectivity have not been implemented yet.

It is our opinion that theoretical and experimental investigations at lower operating temperatures (OTs) are needed, considering that both metal oxide and 2D TMD/MD layered gas sensors are increasingly operated between room temperature and a 100 °C OT. On this account, the amorphous seamless texture of the *a*-SnO_2_ sensor represents an ideal platform for operando DRIFT spectroscopy measurements considering the absence of crystalline planes, an opportunity that rules out any influence of the preparation conditions on the adsorption mechanism of different molecules.^77^

**Figure 9:**
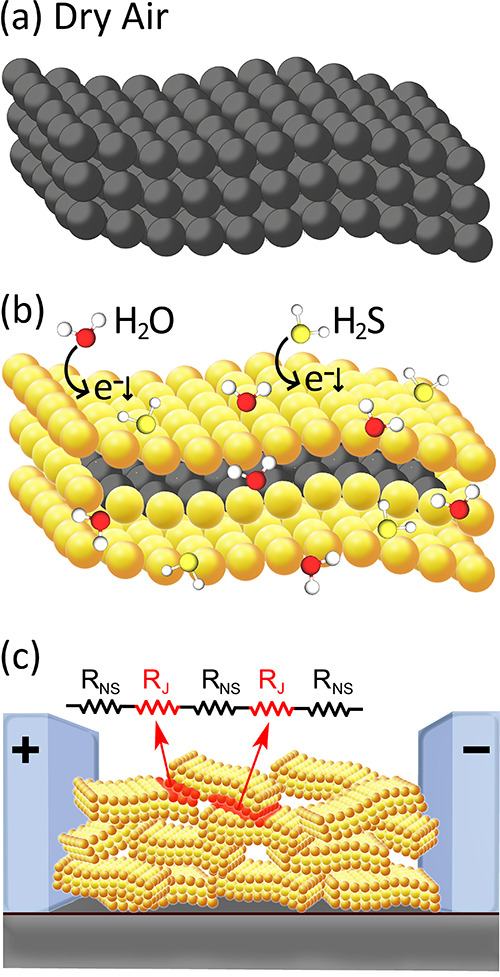
Schematization of a possible gas/water vapor adsorption mechanism over few flakes *a*-SnO_2_ and the conduction mechanism of nanosheet networks. (a) Few-layer *a*-SnO_2_ in dry air; (b) few-layer *a*-SnO_2_ after exposure to H_2_S or H_2_O (yellow regions represent the charge injected zones and the gray inner regions the not injected ones); (c) network morphology of spin-coated *a*-SnO_2_ flakes forming localized intersheet junctions (colored in red) with the schematization of the current transfer equivalent circuit between the sheets (*R*_NS_ = sheet resistance and *R*_J_ = junction resistance).

Figure 9 finally illustrates a possible effect of gas/water adsorption over layered *a*-SnO_2_ and the conduction mechanism of nanosheet networks. Departing from few-layer gray-colored *a*-SnO_2_ flakes, representing the situation in dry air (Figure 9a), as soon as H_2_S gas or H_2_O adsorbs over *a*-SnO_2_, the flake’s surface charge carrier concentration increases, corresponding to the yellow-colored regions of Figure 9b. Depending on the thickness of the stacked flakes, the extension of the injected regions may be limited to surface layers, leaving the inner flakes unaffected (inner gray region of Figure 9b), or eventually extended to the core (fully injected flakes, not shown here). Remarkably, by controlling the liquid-phase exfoliation procedure,^78^ the thickness of the flakes can be easily tailored to cover all the possible conduction regimes, from few-nanometer fully injected thin layers to partially injected thicker ones, therefore effectively modulating the gas response. Lastly, we have reported in Figure 9c the schematization of a spin-coated thin film, comprising a disordered network of *a*-SnO_2_ nanosheets, forming localized intersheet junctions (highlighted in red in Figure 9c), and enabling charge transfer across the layers. In this case, the electrical conduction model is represented as an arrangement of in-series pairs of resistances where *R*_S_ and *R*_J_ represent the sheet and junction resistances, respectively, as recently brilliantly discussed.^79^ Considering that amorphous oxide semiconductors are generally characterized by a high electron mobility (≈10 cm^2^/(V s))^80^ exceptionally topping the field-effect mobility of ≈100 cm^2^/(V s) for amorphous SnO_2_,^81^ a junction-limited conduction mechanism where *R*_J_ ≫ *R*_S_ prevails. This model addresses the formation of Schottky barriers between the flakes, modulated by the nature and composition of the adsorbing gas in the same way as the conduction mechanism of loosely sintered metal oxide nanoparticles in traditional chemoresistive sensors.

## Conclusions

We have reported an innovative and simple procedure to synthesize “LAMOS”, specifically layered amorphous *a*-SnO_2_ metal oxide sensors, by annealing liquid-phase exfoliated 2D SnSe_2_ in air for two weeks at 250 °C. The oxidation process of 2D SnSe_2_ here validated, carried out at temperatures below the crystallization temperature of SnO_2_ (280 °C), enables the spontaneous substitution of sulfur with oxygen atoms in 2D SnSe_2_. Such layered amorphous *a*-SnO_2_ flakes, which are stable up to 250 °C, preserve all the geometrical features of their 2D precursor counterparts. Thin-film sensors of amorphous *a*-SnO_2_ flakes, fabricated by spin-coating over patterned electrodes, are sensitive to H_2_S and humidity at a 100 °C operating temperature, with excellent baseline resistance recovery and sensor’s signal reproducibility over one-year deployment. We also found that the electrical response to H_2_S and humidity of *a*-SnO_2_ is like that of crystalline SnO_2_ microporous metal oxides, with associated humidity cross-effects on H_2_S sensing and a reduced sensor’s signal amplitude with increasing the humidity content. We also demonstrated the hindering effect of water vapor upon H_2_S sensing by a combined DFT+AIMD computational approach, highlighting that both H_2_O and H_2_S compete at the same time, over the same *a*-SnO_2_ adsorption site, according to a dissociative chemisorption mechanism. We additionally indicated a possible conduction mechanism of the *a*-SnO_2_ thin-film device, theorizing the formation of Schottky barriers between the flakes, modulated by the nature and composition of the adsorbing gas, in the same way as the conduction mechanism of loosely sintered metal oxide nanoparticles in traditional chemoresistive sensors.

In conclusion, we have validated an effective strategy to offset typical drift electrical signal phenomena in 2D TMD/MD sensors induced by spontaneous degradation in dry/wet ambient conditions of the sensor’s surface. On this account, we validated a “core” oxidation/amorphization synthesis of pristine 2D SnSe_2_ chalcogenide flakes to yield *a*-SnO_2_ gas sensors. Remarkably, this methodology can be extended to a large variety of TMDs and MCs, opening new opportunities for “LAMOS” interfaces with unexplored surface-science capabilities, probably well beyond gas sensing applications.

## Supporting Information Available

(Section S1) Microstructure, (Section S2) gas sensing, (Section S3) calculation of the low detection limit (LDL) and calibration lines to assess sensitivity, (Section S4) computational methods, and (Section S5) experimental section (PDF)
