# Supplementary material for: Bidimensional Engineered Amorphous a-SnO2 Interfaces: Synthesis and Gas Sensing Response to H2S and Humidity
Source: ACS Sens. 2022 Jun 25;7(7):2058–68. doi: 10.1021/acssensors.2c00887 (PMC9315963; doi:10.1021/acssensors.2c00887)
Supplement: Supplementary file 1 — se2c00887_si_001.pdf [file se2c00887_si_001.pdf]

SUPPORTING INFORMATION

Bidimensional Engineered Amorphous *a*-SnO_2 interfaces: synthesis and gas sensing response to H_2S and humidity

Valentina Paolucci[1,], Jessica De Santis[1], Vittorio Ricci[1], Luca Lozzi[2], Giacomo Giorgi[3,4*] and Carlo Cantalini[1]*

**Corresponding authors:*

Valentina Paolucci (valentina.paolucci2@univaq.it)

Giacomo Giorgi (giacomo.giorgi@unipg.it)

[1] Department of Industrial and Information Engineering and Economics, Via G. Gronchi 18, University of L'Aquila, I-67100 L'Aquila, Italy

[2] Department of Physical and Chemical Sciences, University of L'Aquila, via Vetoio, 67100 L'Aquila (AQ), Italy

[3] Department of Civil & Environmental Engineering (DICA), Università degli Studi di Perugia, Via G. Duranti 93, 06125 Perugia, Italy

[4] CNR-SCITEC, 06123 Perugia, Italy

Section S1 – Microstructure

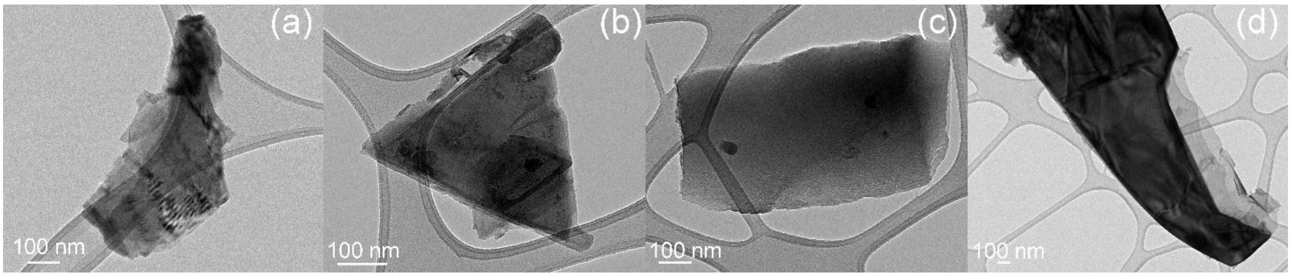

Figure S1. Low resolution TEM images of the as exfoliated $SnSe_2$ flake **(a)** and $SnSe_2$ annealed at: 250 °C 1 week **(b)**, 250 °C two weeks **(c)** and 250 °C two weeks + 280 °C one week **(d)**.

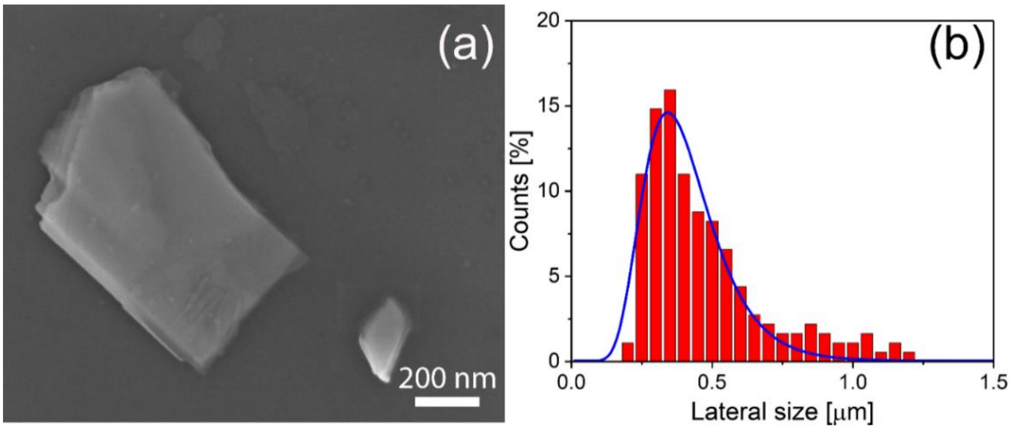

Figure S2. (a) High-resolution SEM of a representative as-exfoliated $SnSe_2$ flake; **(b)** lateral size distribution of the $SnSe_2$ flakes.

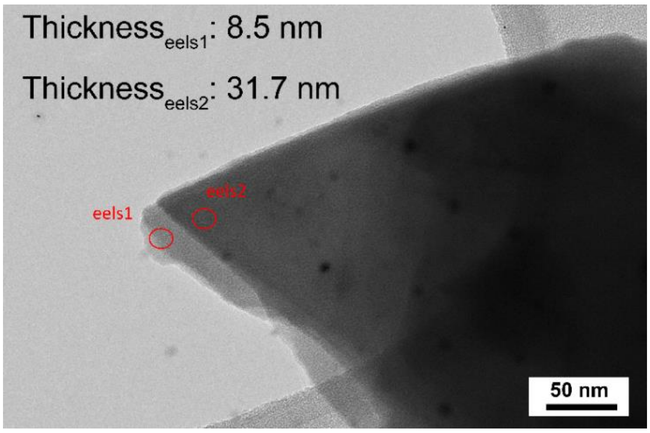

Figure S3. HRTEM of as-exfoliated flakes with thicknesses measured on the red circles by means of EELS technique.

Section S2 – Gas sensing

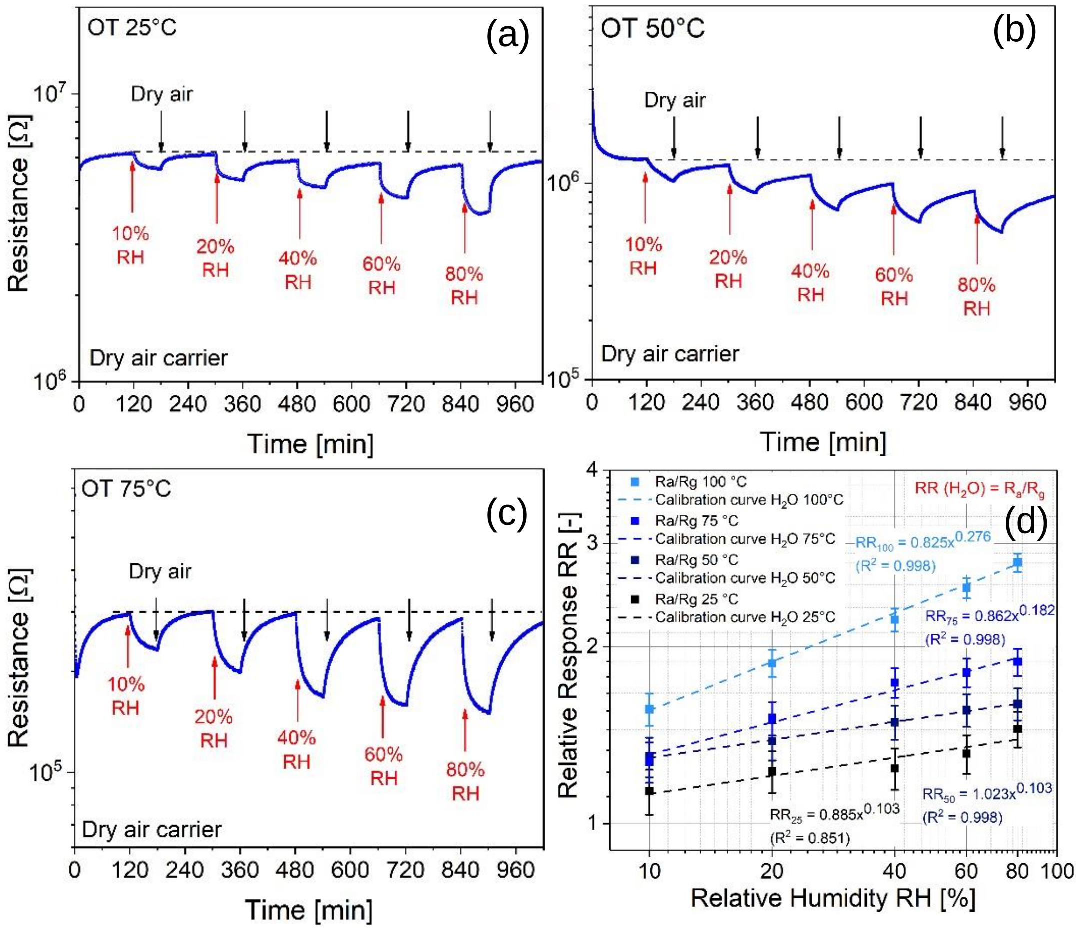

Figure S4. Dynamic electrical responses in dry air at **(a)** 25 °C, **(b)** 50 °C and **(c)** 75 °C OT to H_2O in the range 10-80% RH (all @25 °C); **(d)** log/log calibration plots of Relative humidities from 10% to 80% @ 25°C at 25, 50, 75 and 100 °C OT

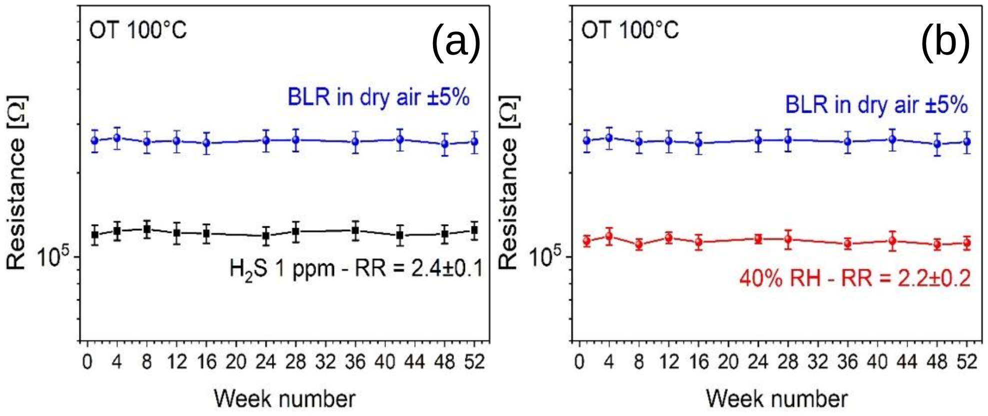

Figure S5. Long-term stability of the resistance to **(a)** 1 ppm H_2S and **(b)** 40% RH @ 25°C over a period one year (with associated standard deviations calculated over a set of 5 consecutive measurements carried at the end of each week.

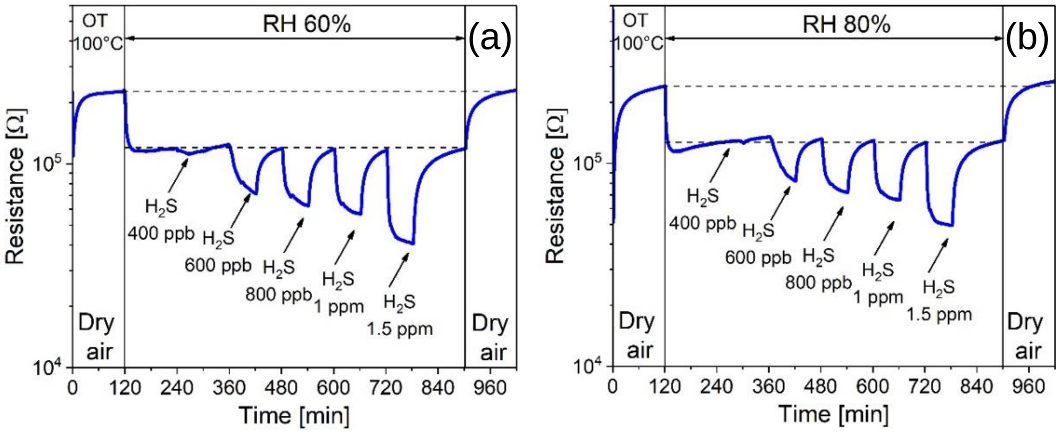

Figure S6. Dynamic electrical responses at 100°C OT to H_2S (400 ppb - 1.5 ppm) with 60% RH **(a)** and 80% RH **(b)** as interfering gases

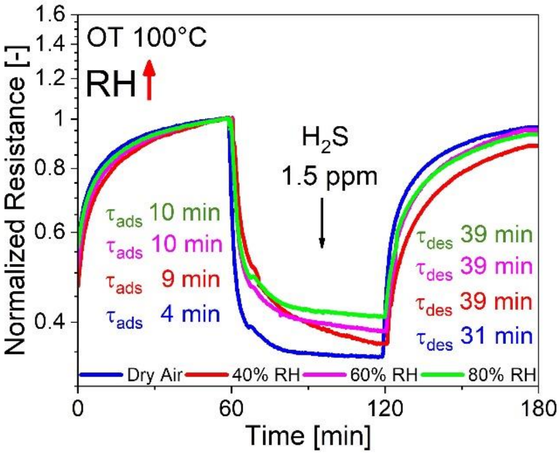

Figure S7. Adsorption and desorption times of 1.5 ppm H_2S calculated at different RH% from 0 to 80% @ 25°C and 100°C OT.

Section S3 – Calculation of the low detection limit (LDL) and calibration lines to assess sensitivity

Gas sensor sensitivity (SS) is represented by the slopes of the calibrating line lines obtained by plotting in a log-log scale $(Rg/Ra)_{H2S}$ as function of increasing H_2S gas concentrations. According to the IUPAC definition [1s], the lowest detection limit (LDL) of a sensor can be estimated starting from experimental data, according to:

$$LDL_{ppm} = 3\frac{rms_{noise}}{slope}$$

where "slope" represents the slope of the linear fitting of the logarithm of relative response vs. the logarithm of the concentration. For the calculation of the rms_{noise}, ten points at baseline (i.e. baseline

before gas exposure) of the sensor were considered and fitted by a fifth order polynomial equation. Then, rms_{noise} was calculated as

$$rms_{noise} = \sqrt{\frac{(R_i - R)^2}{N}}$$

where N is the number of points, Ri are the experimental data points, and R is the calculated value from the fifth order polynomial fitting.

In our case, for H_2S, the noise and slope values of the sensor were 0.04 and 0.55 ppm^{-1} respectively, giving an LDL value of the H_2S sensor of about 0.21 ± 0.1 ppm.

Table S1. Calculated H_2S limit of detection (LOD) of the *α*-SnO2 at different RH content (@ 25°C) and 100°C OT

Test Conditions	H_2S LOD [ppb]
Dry air	210
40% RH	280
60% RH	360
80% RH	380

Section S4 – Computational Methods

Computational procedure & validation of the model

The modeling of the amorphous *a*-SnO_2 NS (Figure S8) has been inspired by that of amorphous TiO_2 ones,[2s,3s] while the anchoring mechanisms of H_2S molecule stem from a chemisorbed and physisorbed (molecular) model, related to H_2S adsorption on stable crystalline rutile SnO_2 surfaces.[4s] Specifically, we considered 4 initial guesses, i.e. chemisorbed H_2S (with one broken H—S bond) on top of: (*i*) a 4-fold and (*ii*) a 5-fold Sn at the surface of a-SnO_2 NS and physisorbed H_2S still on top of a (*iii*) 4-fold and a (*iv*) 5-fold Sn at the surface of *a*-SnO_2 NS. The overall physisorption and chemisorption attack mechanisms are sketched in Figure S8a,b, respectively. Besides the physisorbed configuration mode (a), in the chemisorbed attack (b), the H atom resulting from the H—S bond-breaking of the H_2S molecule saturates an oxygen atom at the surface.

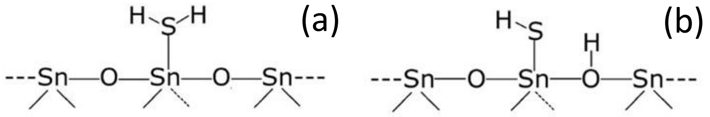

Figure S8. Sketch of the initially considered attacks. H_2S **(a)** physisorbed and **(b)** chemisorbed on the amorphous SnO_2 surface. The two schemes do not consider the coordination, 4-fold or 5-fold, of Sn atom.

Corresponding H_2S molecule's adsorption energies (E_{ads}) on the amorphous surface has been computed according to the following equation:

$$E_{ads} = E_{surf\_H2S} - (E_{surf} + E_{H2S}) \qquad (S1)$$

where E_{surf_H2S}, E_{surf}, and E_{H2S} are the energy of the final system with the hydrogen sulfide molecule anchored on the *a*-SnO_2 NS surface, of a single optimized molecule of H_2S, and of the optimized initial clean *a*-SnO_2 NS respectively. Remarkably, the adsorption energies calculated with Eq. (S1) for (*i-iv)* H_2S anchoring are all quantitatively endothermic (i.e. $E_{ads} > 0$) with the molecularly adsorbed ones (Figure S8a) more unstable than the chemisorbed systems (Figure S8b), partly supporting previously reported data for H_2S adsorbed on SnO_2 rutile (110) surface.[4s] Such unexpected result was further investigated considering the occurrence of other different geometries obtained by thermalizing the initially calculated (*i-iv*) metastable systems at 300 K, by means of short AIMD runs and subsequently re-optimizing some representative trajectories for each system

at the DFT level (0 K, PAW/PBE). Following this procedure, we found three exothermic geometries, all chemisorbed, whose structural features are discussed in the main text. Accordingly, adsorption energies (E_{ads}) shown in Table 3 refer to (i) values obtained by thermalizing (300 K, AIMD) and re-optimizing (0 K, DFT) only products (i.e., precisely the term E_{surf_H2S} only in Eq.(S1)), (ii) values (in brackets) obtained by thermalizing (300 K, AIMD) and re-optimizing (0 K, DFT) for the sake of consistency both products and reactants of the anchoring (i.e., both E_{surf_H2S} and E_{surf} terms in Eq.(S1)). Notably, E_{ads} values, despite being smaller for the latter case, show a consistent trend (exothermic), attesting no significant changes in the adsorption thermodynamics (i.e., by thermalizing or not the clean *a*-SnO_2 at 300 K before the DFT optimization), a result that in this case can be ascribed to a temperature-driven saturation of dangling bonds initially present in the amorphous layers.

As for the case of H_2S, in this study we additionally thermalized water chemisorbed structure with short AIMD runs at 300 K, further re-optimizing at DFT level some trajectories of relevance, yielding no significant structural differences by comparing the combined AIMD+DFT computations with previous DFT ones [5s].

Theoretical setup

Geometry optimization of the free-standing amorphous layer of SnO_2 and of the H_2S//*a*-SnO_2 anchored systems was performed by means of Density Functional Theory (DFT)-based simulations as implemented in the Vienna Ab-initio Simulation Package (VASP) code. [6s–9s] We similarly employed the projector augmented wave (PAW) method [10s] along with the generalized gradient approximation exchange–correlation functional as parametrized by Perdew–Burke–Ernzerhof (PBE). [11s] The DFT-D3 dispersion correction was also used to include the van der Waals interactions [12s, 13s]. We selected plane-wave cutoff energy of 600 eV. All the structures were optimized until the forces on all atoms were smaller than 0.04 eV/Å.

Starting from the initially optimized structure of bulk *a*-SnO_2 (192 atoms, 64 Sn, 128 O, 3 × 3 × 3 Γ-centered *k*-point sampling of the Brillouin Zone (see ref.[5s] for further details) we have added a large amount of vacuum (~15 Å) along the three directions to assemble the nanosheets (NSs) and prevent accordingly spurious interactions among the replicas in the non-periodic directions. We corrected all the slabs for the possible residual dipole still along the non-periodic directions and fully re-optimized both lattice parameters and ionic positions in all the systems investigated, finding that the most stable orientation is the one where the non-periodic direction is oriented along the *c*-axis (see Figure S9). NS in-plane optimized lattice parameters are *a* = 13.17 and *b* = 13.37 Å, respectively. The initial anchoring systems reported in the sketch in Figure S8 are thus optimized at the PAW/PBE level of theory. As mentioned, the initially obtained *i-iv* systems (described in the main text) are all endothermic.

Ab-Initio Molecular Dynamics (AIMD) simulations [14s] have been accordingly performed to better shed light on the anchoring mechanism. In particular, short runs of 5 ps (2.5 ps for equilibration, 2.5 ps for production) have been performed on the initially DFT optimized anchoring geometries in a canonical ensamble (NVT) at T = 300 K and with a timestep of 0.5 fs. [14s] A significant trajectory after 5 ps is thus extracted for each anchored system which is once more fully re-optimized @PAW/PBE. Finally, we have computed the Bader charge distribution associated with H_2S absorption for each structure of interest by means of the Bader code. [15s–18s]

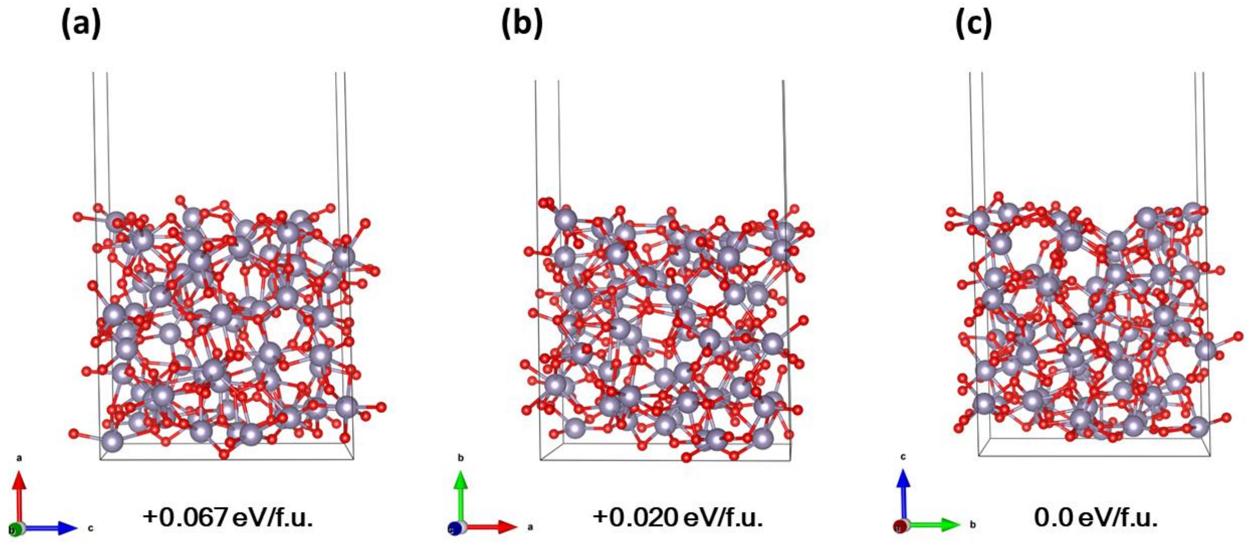

Figure S8. Optimized geometry of the three NSs obtained adding vacuum on top of (a) the (100), (b) the (010), and (c) the (001) direction of the initially optimized bulk *a*-SnO_2. For each structure the energy per formula unit relative -to the most stable one, (c)- is similarly reported. [Mauve: Sn; Red: Oxygen atoms].

Section S5 – Experimental Section

Synthesis of amorphous a-SnO_2

$SnSe_2$ crystals (Ossila – UK) were exfoliated in NMP (N-Methyl-2-pyrrolidone, Sigma-Aldrich) solvent by means of Sonication-Assisted Liquid Phase Exfoliation (LPE). The dispersion was sonicated at 25 °C using a Sonics VC 505 working at 20 kHz and 400 W for 3 hours and subsequently centrifuged to remove the solvent. Flakes were then redispersed in ethanol, spin-coated over Si_3N_4 (at 1500 rpm for 30 s) substrates, and successively annealed in static air at 250 °C for two weeks.

Materials characterization

Microstructure was investigated by SEM imaging (Gemini FESEM 500) working at an accelerating voltage of 2 kV, and by TEM, HRTEM and SAED performed using Jeol JEM 2010F (Jeol Ltd., Tokyo, Japan) operating at 200 kV. Thermogravimetric (TG) and differential thermal (DTA) analyses of exfoliated $SnSe_2$ were carried out in air in Air and in N_2 atmospheres using LINSEIS L81-I at a heating rate at 5 °C/min from 25 °C to 1050 °C. XPS spectra were collected using a PHI 1257 spectrometer equipped with monochromatic Al Kα source (hν =

1486.6 eV) and operating at a spectral resolution of 250 meV. Grazing Incidence X-Ray Diffraction (GIXRD) was performed by XRD-PAN Analytical X'PERT Pro using Cu Kα1 radiation (λ = 1.5406 Å) in parallel geometry using an incident angle of 0.8°.

Gas sensing measurements

Thin films were prepared by spin coating 50 µL of centrifuged dispersion containing 2D $SnSe_2$ nanosheets over Si_3N_4 substrates provided with Pt finger type electrodes (30 micron apart) on front side and heater on backside and mounted inside a Teflon test chamber (500 cm^3) to measure resistance variations (Agilent 34970A). Thin films were in situ annealed at 250 °C in dry air for two weeks to yield amorphous *a*-SnO_2, while in "operando" monitoring the variation of the baseline resistance as previously reported[28]. After baseline resistance stabilization, films were exposed to H_2S and humidity at different concentrations in the temperature range 25–150 °C. Different H_2S concentrations in the range 400 ppb - 1.5 ppm were obtained by diluting in dry air certified H_2S gas mixture (5 ppm in air) using mass flow controllers (MKS 147) with a constant flow rate of 500 sccm/min. Distinct relative humidity's (RH%) were obtained by mixing dry air with saturated water vapor air and RH% content monitored before injection at a temperature of 25 °C (Thermo-Hygrometer AHTD-625). The following definitions were applied in gas sensing measurements: (BLR), Baseline Resistance represented by the sensor's electrical resistance in air at equilibrium; sensor's signal or Relative Response, the relative resistances changes $RR = R_{Air}/R_{Gas}$ for a given concentration of gases; (S), Sensor Sensitivity, the slope of the calibration curve in the log-log sensitivity plot; (τ_{ADS}, τ_{DES}) adsorption and desorption times, the time needed to reach 90% of the resistance value at equilibrium after adsorption and desorption of the analyte.

References

(1s) Currie, L. A. Nomenclature in Evaluation of Analytical Methods Including Detection and Quantification Capabilities (IUPAC Recommendations 1995). *Anal. Chim. Acta* **1999**, *391* (2), 105–126. https://doi.org/10.1016/S0003-2670(99)00104-X.

(2s) Manzhos, S.; Giorgi, G.; Yamashita, K. A Density Functional Tight Binding Study of Acetic Acid Adsorption on Crystalline and Amorphous Surfaces of Titania. *Molecules* **2015**, *20* (2), 3371–3388. https://doi.org/10.3390/molecules20023371.

(3s) Prasai, B.; Cai, B.; Underwood, M. K.; Lewis, J. P.; Drabold, D. A. Properties of Amorphous and Crystalline Titanium Dioxide from First Principles. *J. Mater. Sci.* **2012**, *47* (21), 7515–7521. https://doi.org/10.1007/s10853-012-6439-6.

(4s) Wei, W.; Dai, Y.; Huang, B. Role of Cu Doping in SnO2 Sensing Properties toward H 2S. *J. Phys. Chem. C* **2011**, *115* (38), 18597–18602. https://doi.org/10.1021/jp204170j.

(5s) Paolucci, V.; De Santis, J.; Lozzi, L.; Giorgi, G.; Cantalini, C. Layered Amorphous A-SnO2 Gas Sensors by Controlled Oxidation of 2D-SnSe2. Sensors Actuators B Chem. 2022, 350, 130890. https://doi.org/10.1016/J.SNB.2021.130890.

(6s) Kresse, G.; Hafner, J. Ab Initio Molecular Dynamics for Liquid Metals. *Phys. Rev. B. Condens. Matter* **1993**, *47* (1), 558–561. https://doi.org/10.1103/PHYSREVB.47.558.

(7s) Kresse, G.; Hafner, J. Ab Initio Molecular-Dynamics Simulation of the Liquid-Metalamorphous-Semiconductor Transition in Germanium. *Phys. Rev. B* **1994**, *49* (20), 14251–14269. https://doi.org/10.1103/PhysRevB.49.14251.

(8s) Kresse, G.; Furthmüller, J. Efficiency of Ab-Initio Total Energy Calculations for Metals and Semiconductors Using a Plane-Wave Basis Set. *Comput. Mater. Sci.* **1996**, *6* (1), 15–50. https://doi.org/10.1016/0927-0256(96)00008-0.

(9s) Kresse, G.; Furthmüller, J. Efficient Iterative Schemes for *Ab Initio* Total-Energy Calculations Using a Plane-Wave Basis Set. *Phys. Rev. B* **1996**, *54* (16), 11169. https://doi.org/10.1103/PhysRevB.54.11169.

(10s) Kresse, G.; Joubert, D. From Ultrasoft Pseudopotentials to the Projector Augmented-Wave Method. *Phys. Rev. B* **1999**, *59* (3), 1758. https://doi.org/10.1103/PhysRevB.59.1758.

(11s) Perdew, J. P.; Burke, K.; Ernzerhof, M. Generalized Gradient Approximation Made Simple. *Phys. Rev. Lett.* **1996**, *77* (18), 3865–3868. https://doi.org/10.1103/PhysRevLett.77.3865.

(12s) Grimme, S.; Antony, J.; Ehrlich, S.; Krieg, H. A Consistent and Accurate Ab Initio Parametrization of Density Functional Dispersion Correction (DFT-D) for the 94 Elements H-Pu. *J. Chem. Phys.* **2010**, *132* (15), 154104. https://doi.org/10.1063/1.3382344.

(13s) Grimme, S.; Ehrlich, S.; Goerigk, L. Effect of the Damping Function in Dispersion Corrected Density Functional Theory. *J. Comput. Chem.* **2011**, *32* (7), 1456–1465. https://doi.org/10.1002/JCC.21759.

(14s) Soler, J. M.; Artacho, E.; Gale, J. D.; García, A.; Junquera, J.; Ordejón, P.; Sánchez-Portal, D. The SIESTA Method for Ab Initio Order-N Simulation. *J. Phys. Condens. Matter* **2002**, *14* (11), 2745. https://doi.org/10.1088/0953-8984/14/11/302.

(15s) Tang, W.; Sanville, E.; Henkelman, G. A Grid-Based Bader Analysis Algorithm without Lattice Bias. *J. Phys. Condens. Matter* **2009**, *21* (8), 084204. https://doi.org/10.1088/0953-8984/21/8/084204.

(16s) Henkelman, G.; Arnaldsson, A.; Jónsson, H. A Fast and Robust Algorithm for Bader Decomposition of Charge Density. *Comput. Mater. Sci.* **2006**, *36* (3), 354–360. https://doi.org/10.1016/J.COMMATSCI.2005.04.010.

(17s) Sanville, E.; Kenny, S. D.; Smith, R.; Henkelman, G. Improved Grid-Based Algorithm for Bader Charge Allocation. *J. Comput. Chem.* **2007**, *28* (5), 899–908. https://doi.org/10.1002/JCC.20575.

(18s) Yu, M.; Trinkle, D. R. Accurate and Efficient Algorithm for Bader Charge Integration. *J. Chem. Phys.* **2011**, *134* (6), 064111. https://doi.org/10.1063/1.3553716.
